# Supplementary material for: How, when, and why do inter-organisational collaborations in healthcare work? A realist evaluation
Source: PLoS One. 2022 Apr 11;17(4):e0266899. doi: 10.1371/journal.pone.0266899 (PMC9000100; doi:10.1371/journal.pone.0266899)
Supplement: S1 File — (DOCX) [file pone.0266899.s001.docx]

**Regulator interview guide**

1. Collaboration has had priority in the FYFV and gets strong coverage in the 10 year plan, how far do you think the NHS shifting with respect to attitudes towards collaboration?

2. What do you feel are the main aims of partnership working and collaboration in the NHS? Why partner? Or what is the motivation to do so?

3. (if not covered above) What are some of the main partnership arrangements you have knowledge about? Why were these introduced?

4. What are the main influences on how partnering works in practice? Ingredients that you need for success? The barriers likely to impact? (ind, org, system wide)

5. What about the impact of partnering in relation to organisational and patient outcomes? Do you have any thoughts about that?

6. (If not covered above) What are some unforeseen impacts of partnerships in your experience?

7. Do you have any thoughts about the future of current partnering arrangements and the sustainability of these efforts?

**Interview guide – NHS Leaders/practitioners**

1. How did the partnership you are involved with come to be? What effect has this had on the process of getting it up and running and dealing with the partners?

2. Are there other reasons for the partnership taking place that may not be publicly stated? (unstated drivers).

3. How is the partnership intended to improve your respective organisations?

4. Can you describe the process of setting up the partnership, from the envisioning of the partnership, it to your current situation, and how that went?

5. Have you encountered any major barriers to establishing the partnership? What has helped you overcome these?

6. How has working with the people in the other organisation been?

7. How are attitudes towards the partnership within the workforce? How have these attitudes been affected over time?

8. Has the need to work in partnership created any unforeseen issues?

9. How do you feel about the future of your partnership arrangement? What is happening to organisational performance as a result of the partnership
