# Supplementary material for: How, when, and why do inter-organisational collaborations in healthcare work? A realist evaluation
Source: PLoS One. 2022 Apr 11;17(4):e0266899. doi: 10.1371/journal.pone.0266899 (PMC9000100; doi:10.1371/journal.pone.0266899)
Supplement: S2 File — (DOCX) [file pone.0266899.s002.docx]

Supplementary File 2 – Collaborative Functioning Coding Structure

## Coding structure

| Name |
| --- |
| Deductive |
| Impact of regulation |
| Competition requirements |
| Covid-19 |
| Enforced changes |
| Inspections and CQC |
| Regulation effect on trust |
| Inertia vs. performance |
| Leadership skills |
| Bad qualities |
| Lack of accountability |
| Missing actor |
| Resistance to change |
| Good qualities |
| Commitment |
| Consistency of approach |
| Empathetic leadership |
| Espousing local benefits of collaborating |
| Fostering culture |
| Fostering talent |
| Generosity |
| Learning from others |
| Listening and reacting appropriately |
| Negotiation & diplomacy |
| Pursuasiveness |
| Reframing |
| Removing noncooperative staff |
| Showing vulnerability & humility |
| Visibility |
| Leadership link to culture |
| Mechanisms |
| Clarity and sharedness of vision |
| Clear vision linked to faith |
| Lack of sharedness |
| Shared vision linked to trust |
| Collaborative vs. competitive behaviour |
| Trust and faith preceding collaborative behaviour |
| Confidence in contract |
| Formalisation |
| Conflict |
| Conflict link to faith |
| Conflict link to trust |
| Intra-organisational conflict |
| Conflict resolution and accountability |
| Change in leadership |
| Cultural integration |
| Professional divide |
| Faith & initial faith |
| 'Energy' |
| Capacity |
| Dedicated staff |
| Championing |
| Flexibility |
| Inclusivity |
| Lack of faith |
| Stopping at the right time |
| Overpromising |
| Staff consultation |
| Staff turnover |
| Task complexity |
| Complexity link to faith |
| Voluntary |
| Workforce issues |
| Interpersonal communication and information sharing |
| Communication link to trust |
| Geographical distance |
| Structure to enable communication |
| Perceived legitimacy of collaboration |
| Perception of takeover |
| Sense of loss |
| Service user engagement |
| Perception of progress |
| 'Quick wins' |
| Evaluation and measurement |
| Perception of lack of progress |
| Risk tolerance |
| Trust & initial trust |
| History of prior collaboration |
| Link to trust |
| Mutual understanding |
| Power |
| Reputation of the organisation |
| Respect |
| Sovereignty |
| Autonomy |
| Staff sharing |
| Successes leading to further trust |
| Voluntariness |
| Inductive |
| Commissioning |
| Drivers & entry points |
| Unstated drivers |
| Failure to achieve outcomes and dissolution |
| Initial performance dip |
| Integrated-care items |
| Mandated vs. voluntary |
| Misc. interesting excerpts |
| Participant history with collaboration |
| Structure of collaboration |
| Negatives of collaboration |
| Novel contributions to theory |
| Novel CMOs |
| Impact of population type |
| Peer pressure and perception of progress |
| Outcomes |
| Unintended outcomes |
| Tendency towards greater integration in health systems |
| Typologies of partnering |
