# Supplementary material for: How, when, and why do inter-organisational collaborations in healthcare work? A realist evaluation
Source: PLoS One. 2022 Apr 11;17(4):e0266899. doi: 10.1371/journal.pone.0266899 (PMC9000100; doi:10.1371/journal.pone.0266899)
Supplement: Attachment — Submitted filename: Feedback to authors - PlosOne.docx [file pone.0266899.s003.docx]

**Feedback to authors**

The authors have undertaken a comprehensive examination of interorganisational collaborations. The article is interesting, insightful and particularly timely for IOC stakeholders in England (and likely in other geographies too!). I have provided a few minor suggestions for amendments to a future version of this manuscript below:

Abstract

- The abstract is comprehensive, but I wonder whether you could be clearer about who you interviewed and the data collection period. If there is space in the findings section, you might want also to highlight the contribution of leadership you identified in the data.

Introduction

- L51 I wonder whether the examples of IOCs you reference (*e.g.,* buddying (4), provider alliances (5), Accountable Care Organisations (6), and mergers (7)) could be brought to life a bit more? You could do this by providing the organisation types involved in your cited examples and the purpose or aims of the collaborations?
- L55-57 ICSs were introduced in shadow form in England (not UK) in 2021 and will be statutory from 2022. It may also be worth noting that this new IOC (ICSs) have been derived in a context where policy is moving away from decades of competition and towards collaboration at a range of levels and through a number of routes (e.g. At place level through place-based partnerships and provider collaboratives, as well as at neighbourhood level through primary care networks). You may not have room to add all of this detail, but perhaps could suggest that the policy direction is moving toward collaboration and away from competition, as this sets the context for your findings.
- L60 “The extent to which IOCs deliver the expected benefits to care quality, safety, and efficiency, is contested (12–15)” Would it be more accurate to suggest that the benefits versus the costs – or to mention efforts/costs in some capacity?

Developing an understanding of IOC functioning

- Would there be value in spelling out some of your key CMOCs tested here? The addition of these might make Figure 2 a bit easier to navigate and comprehend.

Evaluation objectives

- L134 I wonder whether you could be clearer that you would like the findings to be used by IOC leaders (as well as policymakers and researchers, if that is the case?).

Recruitment process and sampling

- It would be helpful to know how the case study organisations map out against the full breadth of IOCs you have identified in the literature in your previous work. Are the case studies representative? Are they mostly hospital alliances or hospital-led? For example, were the ICS leads from secondary care provider organisations? You’ll no doubt be aware that there are challenging power dynamics between primary and community, secondary and tertiary providers, and some of that comes out in your quotes, but the context of each of the case studies may need to be elucidated to help interpret findings.
- It would be helpful if you could be more transparent about how many participants you approached, how many agreed/declined, as well as how many were known to the research team in advance.

Sample

- L182 “focus group” not “focus groups”
- Would you be able to indicate which of your participants took part in the focus group in Table 1? It looks like it could have been Alliance 1, but it’s not clear.

Interviews and setting

- L 194-197 You suggest that later interviews used the teacher-learner cycle – could you indicate at which point in data collection you used this and why? You suggest it was mostly with the people who were interviewed twice, but did you also use this approach with others? Transparency around this would help other researchers using this approach while undertaking realist evaluations (and as a bonus might increase citations of your methods).

Findings

- Most of your CMOCs were confirmed, but is there more you could say about where interviewees refuted your propositions? This would strengthen findings.
- Were there any differences between types of IOCs that you think should be mentioned?
- It would be helpful to define initial faith in Table 2 in the same way that you have defined initial trust.
  - And on a related note, is there anything more that could be said about initial faith and initial trust? I ask only because these seem essential to enabling mechanisms (and possibly linked to ‘good relationships’, which I seem to hear about when I speak to the frontline about collaborative endeavours).

Discussion

- Table 3 is a very helpful summary, as will be your practical guide. Is there any way that you could add a URL for the guide to this article (i.e. a link to a webpage with ‘holding information’ asking for people to add their email address to receive the guide when it’s published)? This will save readers having to search for it online and may increase the chance that your research reaches your key audience of IOC leaders in a digestible format.
- L664 “through contracts” not “through contract”

Limitations

- If indeed the sample was more hospital focused, please could you say so?

Conclusions

- L725 “focus group” not “focus groups”
- L726 Delete “The evaluation included 32 interviews to better understand IOCs.” and instead add information about 32 interviews and 1 focus group to the line above. This may avoid confusion about whether the focus group helped confirm the CMOCs.
