# Supplementary material for: How, when, and why do inter-organisational collaborations in healthcare work? A realist evaluation
Source: PLoS One. 2022 Apr 11;17(4):e0266899. doi: 10.1371/journal.pone.0266899 (PMC9000100; doi:10.1371/journal.pone.0266899)
Supplement: Attachment — Submitted filename: ResponseToReviewers_PLOSOne.docx [file pone.0266899.s004.docx]

Dear Dr. Mahmoud and reviewers,

We thank the editor and reviewers for all their comments which have strengthened the manuscript greatly. We have also taken the opportunity to go through and ensure proper use of language throughout. We have responded to the reviewer comments in the table below, in which the line numbers we cite refer to the lines in the version of the document with tracked changes.

| Reviewer comments | Response to reviewer |
| --- | --- |
| Reviewer 1 | |
| 1. Line 100: The authors could reiterate their rationale for claiming that moving from competitive behaviour towards collaboration is ‘ideal’ given the historically contingent nature of this claim. While it's illustrated in the programme theory diagram, this is important because this claim goes on to inform language choices throughout the piece. For example, the authors regard leadership behaviours that are conducive to collaboration as ‘positive’ (line 283) vs those that aren’t which are ‘negative’ (363). Organisations are described as moving ‘back towards competing’ (329) implying regression. The authors could consider either justifying their choice of absolute terms, or using alternative terms that makes their contingency explicit. | Thank you for all of your comments which have strengthened this manuscript.  We did not necessarily mean to insinuate that collaboration was ‘ideal’ from a policy point of view, rather that it was ‘ideal’ from a collaboration point of view where collaboration is already the end-goal. We have clarified this throughout the manuscript where we have used such language (e.g. line 134). |
| 2. Line 180 onwards: the authors mention the use of a focus group attended by eight ‘participant representatives’. It’s unclear what a ‘participant representative’ is, and only methods and participant details are described further for interviews. Further information about the focus groups should be included for completeness. | We have added significant further information about the focus group attendees to the *Sample* section. |
| 3. Line 244: the link between the quotation and the surrounding text should be clarified – it’s not obvious how it is relevant to the analysis. | Thank you, we have now clarified the context for the quote further in the text (line 321). |
| 4. Line 294: the link between the quotation and the text before it should be clarified – the authors seem to be equating empathetic leadership with support and humility but the connection isn’t obvious. | Thank you, we have added a different quote here to better support the text in this section (line 381). |
| 5. Line 304: given the authors mention that their interviews moved online during the pandemic (200), they could consider whether the significant changes to how people work globally impacts on the finding the communication is adversely affected by geographical difference. Relatedly, to ensure the article is as current as possible, the authors could acknowledge somewhere in the paper how some of the significant changes to working practices that have resulted from the COVID pandemic might impact upon the utility of their action statements. | We did have significant amounts of interview data devoted to the topic of COVID-19 which, as a context, did impact a number of these mechanisms. It also became clear that different organisations had experienced the pandemic differently. Interviewees talked about it stimulating collaboration in some areas and inhibiting it in others, particularly around communication, where they largely felt that the loss of informal communication typical of face-to-face meetings affected relationship building. However, we felt that incorporating all of the COVID-19 related data into this manuscript could have made it even more lengthy and perhaps taken over to a large degree, making the theory less applicable to non-pandemic circumstances.  However, as this was an important contextual element to include to some readers, we have added some discussion of COVID-19 and some key findings from the interviews in the discussion section for those who might have been interested in the key impacts of the pandemic. |
| 6. Line 316-317: the authors should consider their use of the term altruistic – typically altruism doesn’t involve getting something back later in the way implied, so a different word might be more appropriate. | Thank you, we have changed this passage to reflect more that organisations must expose themselves to vulnerability from another organisation (hence the risk), rather than using this word (line 411). |
| 7. Line 393: The connection between the quotation and the derivation of the new CMOC could be clarified as the quotation itself could be read as commenting the diversity of the population rather than the particular demographic group identified. | We have clarified the context for this quote further to link it better to the CMOC. |
| 8. Line 462: The authors could explain what they mean by ‘infrastructure’ – the quotation appears to be about the codification of key governance processes for which ‘infrastructure’ seems too broad a term. | We have updated this to specifically refer to ‘governance structure’ as opposed to ‘infrastructure’, we hope this is clearer for the reader (ln 621). |
| 9. Line 505-6: the first two sentences under ‘cultural integration’ appear to be inconsistent. If culture is being defined as shared behaviours and attitudes (i.e., an emergent property of social relations), then saying it is ‘essential to organisational success’ doesn’t quite work. They authors should clarify what they mean here. | We have clarified this as we meant to say ‘collaborative success’ rather than ‘organisational success’ and that these behaviours and attitudes can be more or less open to collaboration (ln 688). |
| 10. Line 570: this sentence appears to be incomplete. | Thank you for flagging this up; this sentence has been updated to better reflect the link to the next section. |
| 11. Line 575: the connection between the quotation and the surrounding text is unclear. The quotation appears to describe the relationship between accountability and conflict within an organisation, so it would be helpful to clarify how this fits into the theory for IOCs. | We have expanded the conflict resolution section with further quotes. It was challenging to get the balance right in terms of content due to the length of the paper, but we have now added a further quote and further explanation of how these quotations link into the theory. |
| 12. Line 593-4: the phrasing of the sentence starting “In England” could be clarified – are the authors talking about a particular model of collaboration that involves privatisation or a more general point? If the former, this could do with a reference. | We have added a reference to support Integrated Care Systems being linked to fears of privatisation in England. |
| 13. Line 701: the authors could clarify what they mean by “genuine” in relation to interpersonal relationships and be explicit about the qualities they are referring to. | We have added the qualifier that ‘genuine’ refers to relationships built upon mutual trust (ln 1010). |
| 14. Some of the references appear to be incomplete | Thank you, we have been through and identified some references that indeed had some information missing, and we have corrected them. Thanks for all your contributions which have helped to strengthen the manuscript. |
| Reviewer 2 | |
| The abstract is comprehensive, but I wonder whether you could be clearer about who you interviewed and the data collection period. If there is space in the findings section, you might want also to highlight the contribution of leadership you identified in the data. | Thank you for all of your suggestions throughout the paper. We have updated the abstract to include these key elements. |
| L51 I wonder whether the examples of IOCs you reference (e.g., buddying (4), provider alliances (5), Accountable Care Organisations (6), and mergers (7)) could be brought to life a bit more? You could do this by providing the organisation types involved in your cited examples and the purpose or aims of the collaborations? | We have added further detail to table 1 about what sectors of organisation are involved in each of the case study IOCs. Hopefully that adds more useful context.  We did ask in many of the interviews what the purpose/aims were of the collaborations, however, these were usually the same list of virtuous undertakings ‘i.e, improving efficiencies, reducing variation in care’, etc. This occurred despite differing IOC forms that were chosen. This made it difficult to form any CMOCs around drivers as a context for collaboration, as there didn’t seem to be any alignment between IOC form and aims of a collaboration. Hence, we decided to focus more on underlying characteristics of collaboration to integrate with theory, i.e., were they mandated, or where were they on the scale of integration, such as mergers vs. buddying.  We have added discussion of this element to the limitations section. |
| L55-57 ICSs were introduced in shadow form in England (not UK) in 2021 and will be statutory from 2022. It may also be worth noting that this new IOC (ICSs) have been derived in a context where policy is moving away from decades of competition and towards collaboration at a range of levels and through a number of routes (e.g. At place level through place-based partnerships and provider collaboratives, as well as at neighbourhood level through primary care networks). You may not have room to add all of this detail, but perhaps could suggest that the policy direction is moving toward collaboration and away from competition, as this sets the context for your findings. | Thank you, we have updated this to rightfully reflect that it applies to England, not the whole UK. We have added further detail on the policy context to the introduction. |
| L60 “The extent to which IOCs deliver the expected benefits to care quality, safety, and efficiency, is contested (12–15)” Would it be more accurate to suggest that the benefits versus the costs – or to mention efforts/costs in some capacity? | Thank you for this comment. We think the following sentence which states that implementing IOCs “involves significant time and effort to establish and run before any benefits may be realised” reflects the cost. However, we have added financial resource to this list of potential costs (ln 97). |
| Developing an understanding of IOC functioning:  Would there be value in spelling out some of your key CMOCs tested here? The addition of these might make Figure 2 a bit easier to navigate and comprehend. | Thank you for the suggestion; we thought about how to address this comment but feel it would be difficult without adding substantially to the length of the manuscript or duplicating what we have already published elsewhere.  As such, we have added another supplementary file (supp file 2) which shows our coding structure in NVivo for this portion of the project, which we hope sheds additional light on how CMOCs were tested and we hope this goes some way to addressing your concerns. |
| Evaluation objectives:  L134 I wonder whether you could be clearer that you would like the findings to be used by IOC leaders (as well as policymakers and researchers, if that is the case?). | We have clarified who the findings might be useful for, including policymakers, researchers, and those implementing IOCs (ln 190). |
| Recruitment process and sampling   - It would be helpful to know how the case study organisations map out against the full breadth of IOCs you have identified in the literature in your previous work. Are the case studies representative? Are they mostly hospital alliances or hospital-led? For example, were the ICS leads from secondary care provider organisations? You’ll no doubt be aware that there are challenging power dynamics between primary and community, secondary and tertiary providers, and some of that comes out in your quotes, but the context of each of the case studies may need to be elucidated to help interpret findings. - It would be helpful if you could be more transparent about how many participants you approached, how many agreed/declined, as well as how many were known to the research team in advance. | 1. The majority of our interviewees were on the acute/hospital side of the IOCs. We have added a statement to the limitations section to flag the impact of this on the power dynamic and our findings to readers. We have added to table 1 information on how the IOC case studies were comprised of different organisations and clarified on line 147 that most of our interviewees were on the acute care side of the IOCs. While this was not intentional, it may have been a result of the pandemic or from where we snowballed from, due to having to move towards contacts known already to members of the team when COVID began and recruitment became very difficult. We have added more information to the limitations section regarding these issues (line 1050 onwards). 2. We have added numbers to the ‘sample’ section for how many interviewees were already known to the team – these often were the starting point for our ‘snowball’ methodology. However, due to the convenience sample and realist methodology we did not record all of the people we contacted as we were not overly concerned about sampling bias. We were principally trying to obtain as representative a sample of case studies as possible as the first priority. Some individuals were approached directly and others were recruited by approaching their organisation R&D departments. Most organisations did not respond. |
| Sample   - L182 “focus group” not “focus groups” - Would you be able to indicate which of your participants took part in the focus group in Table 1? It looks like it could have been Alliance 1, but it’s not clear. | We have corrected line L182 and added further explanation of who the patient representatives were to the *Sample* section. |
| Interviews and setting   - L 194-197 You suggest that later interviews used the teacher-learner cycle – could you indicate at which point in data collection you used this and why? You suggest it was mostly with the people who were interviewed twice, but did you also use this approach with others? Transparency around this would help other researchers using this approach while undertaking realist evaluations (and as a bonus might increase citations of your methods). | Thank you for the suggestion and we agree this could have used more explanation. We have added further information on how we used the teacher-learner cycle in this study and why. This can be found on lines 270-273. |
| Findings   - Most of your CMOCs were confirmed, but is there more you could say about where interviewees refuted your propositions? This would strengthen findings. - Were there any differences between types of IOCs that you think should be mentioned? - It would be helpful to define initial faith in Table 2 in the same way that you have defined initial trust.   - And on a related note, is there anything more that could be said about initial faith and initial trust? I ask only because these seem essential to enabling mechanisms (and possibly linked to ‘good relationships’, which I seem to hear about when I speak to the frontline about collaborative endeavours). | 1. We did not identify any areas that were significantly different to our prior CMOCs. This might be a reflection of having incorporated 86 papers over multiple stages of refinement prior to also refining it with the interview data. It would have been interesting to have explored any refutations in greater depth to understand why these may have occurred, but unfortunately we did not identify any data to allow us to do so. 2. We tried to approach the differences between IOCs by their core characteristics, i.e., by degree of integration, whether the IOC is cross-sector or not, and whether it is mandated or not. It was otherwise difficult due the sample size to make any definitive judgements about whether certain differences in CMOC may be due to IOC structure or other factors (i.e., the causality was unclear). We have clarified this in the limitations section. 3. We have added a definition of initial faith to table 2, drawing attention to the fact that the concept is very much linked to ‘perception of task complexity’ which serves to set expectations for the IOC. |
| Discussion   - Table 3 is a very helpful summary, as will be your practical guide. Is there any way that you could add a URL for the guide to this article (i.e. a link to a webpage with ‘holding information’ asking for people to add their email address to receive the guide when it’s published)? This will save readers having to search for it online and may increase the chance that your research reaches your key audience of IOC leaders in a digestible format. - L664 “through contracts” not “through contract” | We will be releasing our practical tools, including a survey to assess collaborative readiness, alongside the final report. We have signposted this in the discussion to hopefully point readers to that final report (coming in the next few months, hopefully).  We have updated L664, thank you. |
| Limitations   - If indeed the sample was more hospital focused, please could you say so? | We would agree that it was, and have added discussion of this in the limitations section. |
| Conclusions   - L725 “focus group” not “focus groups” - L726 Delete “The evaluation included 32 interviews to better understand IOCs.” and instead add information about 32 interviews and 1 focus group to the line above. This may avoid confusion about whether the focus group helped confirm the CMOCs. | Thank you, we have fixed these errors. Thanks again for all your comments throughout the manuscript. |
| Reviewer 3 | |
| I have some specific comments about the paper – a few of which ask whether there is any additional data to strengthen the points being made – albeit recognising the challenge of word count! | Thank you for your comments which have helped to improve the manuscript. |
| 1) There is an imbalance between the various themes presented. For example the long and detailed presentation on Trust is followed by a short section on risk tolerance. Both themes are recognised to be important in shaping success or failure but much less is said about the latter. And, while the authors rightly point out that one or other party in a collaboration has to give some ground, they haven’t touched on other important aspects of risk taking – such as when board members have to approve risks in the interpretation of regulatory constraints or financial controls– without which various past attempts to collaborate have failed. Are there any other data on risk that could add further insights on this topic? The section on capacity felt similarly weak compared to other sections. | Balancing the amount of content in each section was a challenge given the length of the manuscript. We gave the most attention to those mechanisms that are closest to collaborative behaviour in the causal chain. However, we agree that more detail and context is always useful! So, we have added further quotes and elucidation to bolster the capacity section. For risk, our conceptualisation of it is very much about how organisations must take on risk to build trust with partners in an inter-organisational manner and less about risks that are intractable to collaborating on an intra-organisational level. Hopefully that makes sense. We have added more quotes and explanation to the risk tolerance section to further bolster these findings and clarify what risk tolerance means in this theory. |
| 2) Lines 286 – 300 provide some good data on the challenges and techniques of good leadership  3) The section on task complexity and scale is interesting and well presented with good use of quotes.  4) The section on culture rang true and seems very pertinent for an English audience given recent mergers of CCGs, and the formation of primary care networks. | Thank you, it is great to hear that these sections resonated with you. |
| 5) There is reference in conclusion to regulator perspectives but only one regulator quote is presented (from NMC) and that related to leadership. oIt would be useful to have more data to demonstrate regulators views. | While writing up the manuscript we relied most heavily on those implementing IOCs as those interviews did shed the most direct light on how collaborative functioning works. We also included this already as limitation of the analysis in the limitations section. However, we did already include quotes from interviewees 11, 06, 07, 15, 04, and 24 in the text and table 2 – who were all part of the regulatory/policymaker component of the sample. |
| 6) Two of the action statements in table 3 are unclear / hard to understand. Specifically, the action point on risk seems to be about trust rather than risk and the second bullet point on managing mandated collaborations. | Thank you for pointing this out, we have updated the risk tolerance action statement and the other that you have pointed out for clarity. |
| Overall, I think this paper adds new insights about collaborative working (albeit that some bits could be strengthened ). It is clearly written and meets the standards of the Rameses II guidelines and I recommend publishing it. The editors will need to decide whether to request some additional data to strengthen the sections on risk and on capacity and on regulatory perspectives. | Thank you again for your most helpful feedback. |
| Reviewer 4 | |
| I really enjoyed reading this paper and find it hard to fault!  It is topical with the emergence of ICSs/ICPs and within these primary care networks in the English NHS. It builds nicely on an already published realist synthesis, using well-described realist methods. The paper draws on a wide range of stakeholders for the interviews, although it would have been nice to also see input from stakeholders in Primary Care Networks too. | Thank you for your kind words regarding the paper. We agree that it would have been good to have more voices from other sectors such as PCNs, but this aspect of recruitment was most likely impacted by COVID. We have added discussion of the limitations of our sample to the limitations section. |
| Just before presenting table 2 it would be helpful for the main text to explain that the ‘novelty’ and ‘affirmation’ relate to the middle range theory from the realist synthesis - currently the words new and existing are used. | We have added this qualifier to the column header in table 2. |
| I got a bit confused how the mechanisms listed in the first column of table 2 then relate to the subheadings after. Could this be made clearer? Is there a subheading missing which is in the table? Could the mechanisms and subheadings be numbered? | We have now added a section after table 2 to explain which mechanisms are discussed – which are all of those in table 2 except for ‘competitive vs collaborative behaviour’, which is typically an outcome for the ‘collaborative functioning’ portion of the causal chain. We have also changed the levelling of the headings which could have also reduced clarity.  Thank you again for all of your feedback. |

We look forward to hearing back about the manuscript in due course.

Yours sincerely,

Justin Aunger and authors.
